# Prevalence of the prothrombin G20210A mutation among ischemic stroke patients

**DOI:** 10.34172/jcvtr.2020.39

**Published:** 2020-09-03

**Authors:** Salar A. Ahmed, Sazgar A. Hameed, Bashdar M. Hussen, Abbas Salihi

**Affiliations:** ^1^Department of Clinical Biochemistry, College of Medicine, Hawler Medical University, Erbil, Iraq; ^2^Central laboratory, Hawler Teaching Hospital, Erbil, Iraq; ^3^Department of Pharmacognocy, College of Pharmacy, Hawler Medical University, Iraq; ^4^Department of Biology, College of Science, Salahaddin University-Erbil, Erbil, Iraq; ^5^Department of Medical Analysis, Faculty of Science, Tishk International University, Erbil, Iraq

**Keywords:** Ischemic Stroke, Prothrombin Gene Mutation, Venous Thrombosis

## Abstract

***Introduction:*** Ischemic stroke is characterized as a sudden neurological deficit attributed to an acute focal injury of the central nervous system by a vascular cause. This study was performed to determine the frequency of G20210A mutation in the prothrombin gene and its effectiveness on the incidence of ischemic stroke in the Erbil city of Kurdistan region, Iraq.

***Methods:*** A total of 50 patients with ischemic stroke was analyzed for the detection of prothrombin gene mutation (G20210A), using polymerase chain reaction (PCR), Restriction fragment length polymorphism (RFLP) with Hind III restriction enzyme.

***Results:*** We observed no evidence of an association between ischemic stroke and G20210A mutation in the prothrombin gene in this region.

***Conclusion:*** Our finding demonstrates that prothrombotic gene variant seems not to be linked to the incidence of ischemic stroke in Erbil region.

## Introduction

Stroke is a severe and life-threatening disease that affects the arteries leading to and within the brain, and it’s the foremost cause of long-term disability and a third major cause of mortality and morbidity worldwide.^1^ It occurs when blood flow to an area of the brain is cut off. When that occurs, brain cells are deprived of oxygen and nutrients; as a result, irreversible pathological *damage* to*brain tissue* occurs within minutes of cerebral hypoxia.^2^ The acute disturbance is usually caused by a clot blocking a blood vessel or by a ruptured blood vessel. When brain cells die during a stroke, the abilities controlled by the affected area of the brain such as memory, speech and muscle control will be lost.^3^ The main risk factors for ischemic stroke are age, sex, family history, race, hypertension, hyperlipidemia, diabetes mellitus, obesity, smoking and genetics factors.^4,5^

Prothrombin is a 72 kDa vitamin K-dependent glycoprotein formed in the liver,^6^ it’s a precursor of the serine protease thrombin and play a significant role in the process of hemostasis and thrombosis.^7^ Prothrombin gene mutation G20210A is a common prothrombotic single-nucleotide polymorphism.^8^ The polymorphism is located in a noncoding region of the prothrombin gene, replacing guanine (G) with adenine (A) at nucleotide position 20210 in the 3′-untranslated region of the prothrombin gene.^9,10^ The G20210A polymorphism is associated with high levels of d-dimer^11^ and elevated plasma prothrombin concentrations and approximately a three-fold increased risk of thrombosis, atrial fibrillation,^12^ myocardial infarction^13^ and cerebral venous thrombosis.^14,15^ This mutation has not been detected among Kurdish patients to date. Therefore, to fill this gap, we aimed to investigate the prevalence of prothrombin gene G20210A mutation in incidences of ischemic stroke in Erbil city.

## Materials and Methods

### Patient specimens

The cohort of this study comprised 50 patients (29 males and 21 females) with documented ischemic stroke at the neurology department of Rizgary Teaching Hospital, Erbil. The whole blood (5 mL) for analysis of prothrombin gene mutation was collected into EDTA-anticoagulated vacutainer tubes; the samples were either used immediately for DNA isolation or kept at about -20°C until further analysis. The inclusion criteria of this study are patients with ischemic stroke, aged between 35-82 years, while the exclusion criteria are autoimmune diseases, including antiphospholipid syndrome and a reduction in the functional activity of antithrombin III.

### Detection of prothrombin (G20210A) mutation

#### DNA extraction

Five milliliters of the peripheral venous blood sample was collected from all the study patients. GeNet Bio DNA extraction kit (GeNet Bio, Korea) was used for the isolation of genomic DNA from the blood samples, and the purity and the concentration of the extracted DNA samples were measured by NanoDrop® ND-1000 Spectrophotometer (Thermo Fisher Scientific, Germany) and checked by 1% agarose gel electrophoresis.^16^

#### PCR/RFLP analysis

The mutation analysis for prothrombin gene G20210A was assessed by polymerase chain reaction (PCR). Genomic DNA was used as a template to amplify the interested region of prothrombin gene (345 bp fragment in exon 14 of prothrombin gene) by using a forward primer (5’-TCTAG AAAC AGTTGCC TGGC-3’) and reverse primer (5’-ATAGCA CTGGGAGC ATTGAAGC-3’).

A manual PCR was used with a total reaction volume of 50 µL containing both the forward and reverse primers for prothrombin gene (1 µL) and 25 µL of master mix (2X) which contain (Taq polymerase, deoxynucleotide triphosphate, PCR buffer). A volume of 50 µL of the reaction mixture was aliquoted into each tube, and 4 µL (60 ng) of isolated genomic DNA was added. The samples were amplified on the thermal cycler starting with an initial denaturing phase at 94°C for 5 minutes before amplification, followed by 25 cycles of 94^o^C for 30 seconds, 55^°^C for 1 min, 72^°^C for 2 min, with a final extension period of 5 min at 72^°^C according to manufacturer recommendations. Then, the PCR products were confirmed by 1% (w/v) agarose gel.^17^

The G20210A mutation was detected by digestion of PCR amplified fragment (345 bp) with Hind III digestion enzyme (Promega, USA). The restriction digestion was performed in a total volume of 20 µL: 5 µL PCR product, 10.6 µL dH_2_O, 20 units/2 µL Hind III enzyme, acetylated BSA 0.4 µL and 2 µL buffer. Then, the samples were incubated for 3 hours at 37^○^C, and restriction fragment size analysis was achieved by visualization of digested PCR products after separation by 1% (w/v) agarose gel electrophoresis at 120v current for 75 minutes and stained with ethidium bromide. The enzymatic digestion generates a couple of fragments (322 bp and 23 bp) from the mutant allele and one fragment of 345 bp from wild-type allele.^18^

## Results

### Characteristics of the study population

A total of 50 ischemic stroke patients were included in this study, consisting of 29 males and 21 females with the mean age 58 years, ranging from (35-82) years of age. The incidence of family history of ischemic heart disease and stroke were 32% and 44% respectively. The baseline characteristics of the study are presented in Table 1.

**Table 1:** Baseline characteristics of the study groups of ischemic stroke patients

**Baseline characteristics**	**Patients (n=50)**
Sex (males/females)	29/21
Age (range)	35-82
Age (mean±SE)	58±1.4
Family history of stroke	16 (32%)
Ischemic heart disease	22 (44%)
Hypertension	42 (84%)
Diabetes mellitus	16 (32%)
Prothrombin G20210A mutation	0 (0%)

### PCR/RFLP analysis for amplification of 345 bp sequences of exon 14 of the prothrombin

In the present study, genomic DNA was isolated from studied subjects with the purity (1.7-2.2), the mean value was 25.8 ng/µL, with a range of variation 7.2-55.2 ng/µL.

**Figure 1:**
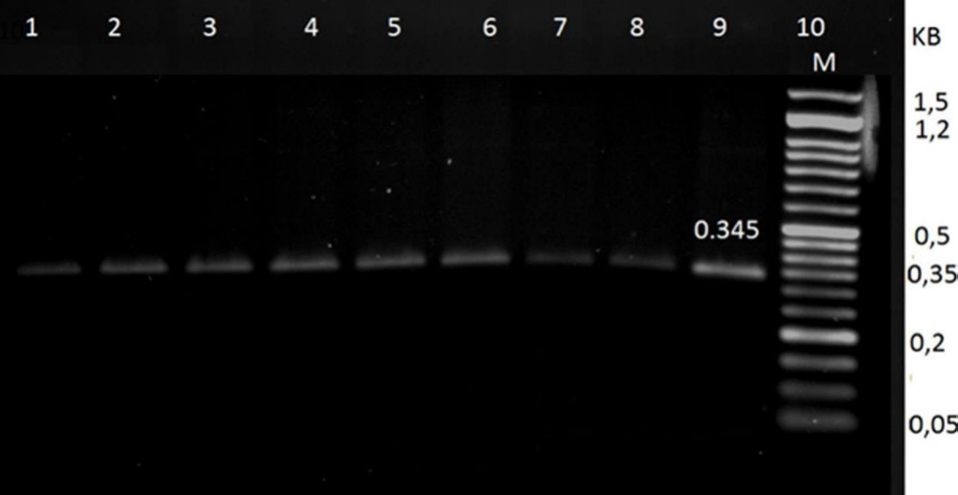
Agarose gel analysis of PCR products corresponding to 345(bp) fragment of Prothrombin gene. DNA markers are shown on the right (Kb). Whereas lines from 1to 9 correspond to PCR products of the amplified DNA from 9 different samples of ischemic stroke patients

**Figure 2:**
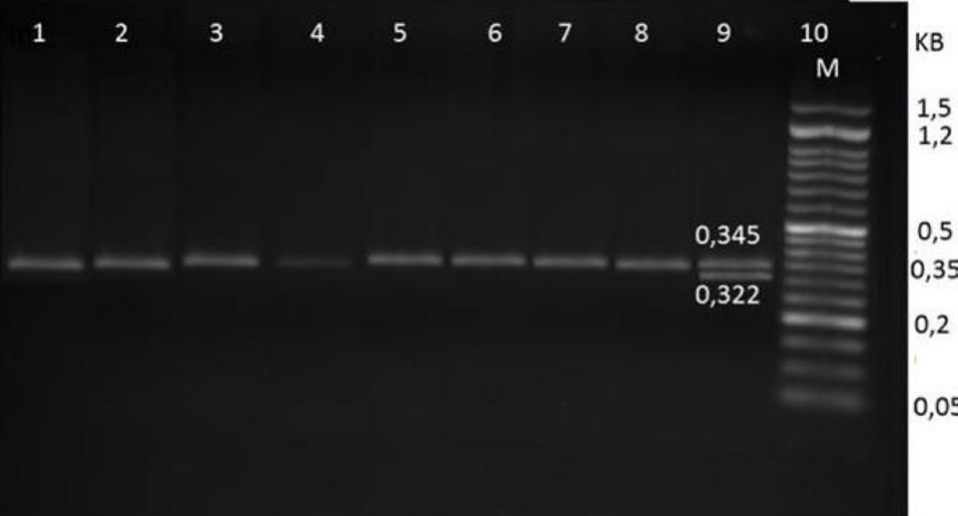
Agarose gel electrophoresis pattern of some RFLP products of Prothrombin gene. Line 10 (M) indicates DNA markers with a 50 bp. Line no. 9 control positive for prothrombin gene mutation (G20210A), the other lanes are negative and did not have the (G20210A) mutation of Prothrombin gene

A 345 bp fragment from exon 14 of the prothrombin gene among randomly selected patients with ischemic stroke was successfully amplified with the specific primers containing Hind III restriction sites, as shown in Figure 1. Furthermore, the results showed that the variant gene allele of prothrombin polymorphisms was not detected in ischemic stroke patients, as shown in Figure 2.

## Discussion

During the last decade,thromboembolic diseases are among the most common causes of morbidity, incapacitation and death, with a general incidence of one in 1000 individuals annually. A single-point mutation in the prothrombin G20210A gene is linked to augmented prothrombin levels and increased risks of venous thrombosis.^19^

Clinical interest in genetic causes of thrombosis has dramatically increased that’s why in the frame of this study, we investigated evidence of the association between G20210A prothrombin gene variant and ischemic stroke in our region. To our knowledge, this study will be the first molecular study in Kurdistan region of Iraq, no previous comprehensive information and studies have been done about G20210A mutation of prothrombin gene and influence, increased risk, and pathogenesis of cerebral infarction among patients suffering from ischemic stroke. When we analyzed the G20210Amutation in the prothrombin gene, among the 50 ischemic stroke individuals, we observed that the G20210Amutation was absent among the study subjects. The absence of a genetic risk factor for ischemic stroke among Kurdish people may be due to the difference in the geographical location and ethnic background.^9^

In conclusion, the G20210A prothrombin gene variant is not associated with increased risk, incidence and pathogenesis of ischemic stroke in Hawler city. Therefore, this study recommends that the genotype documentation of prothrombin gene G20210A mutation is not desirable for patients with ischemic stroke in this region. The limitation of this study is a low sample size. Therefore, we recommend to increase the number of patients and including other prothrombin related gene.

## Competing interests

None declared.

## Ethical approval

Ethics approval was granted by the Research Ethics Committee of the Medical Research Center, Hawler Medical University (4/2019) and informed consent was obtained before phlebotomy.

## Funding

None.
